# Coronary calcium scores on dual-source photon-counting computed tomography: an adapted Agatston methodology aimed at radiation dose reduction

**DOI:** 10.1007/s00330-022-08642-5

**Published:** 2022-03-01

**Authors:** Niels R. van der Werf, Marcel J. W. Greuter, Ronald Booij, Aad van der Lugt, Ricardo P. J. Budde, Marcel van Straten

**Affiliations:** 1 Department of Radiology & Nuclear Medicine, Erasmus MC - University Medical Center Rotterdam, Rotterdam, The Netherlands; 2 Department of Radiology, University of Groningen, University Medical Center Groningen, Groningen, The Netherlands; 3 Department of Robotics and Mechatronics, University of Twente, Enschede, The Netherlands

**Keywords:** X-ray computed tomography, Calcium, Coronary vessels, Imaging phantoms, Radiation dosage

## Abstract

**Objectives:**

The aim of this study was to determine mono-energetic (monoE) level–specific photon-counting CT (PCCT) Agatston thresholds, to yield monoE level independent Agatston scores validated with a dynamic cardiac phantom. Also, we examined the potential of dose reduction for PCCT coronary artery calcium (CAC) studies, when reconstructed at low monoE levels.

**Methods:**

Theoretical CAC monoE thresholds were calculated with data from the National Institute of Standards and Technology (NIST) database. Artificial CAC with three densities were moved in an anthropomorphic thorax phantom at 0 and 60–75 bpm, and scanned at full and 50% dose on a first-generation dual-source PCCT. For all densities, Agatston scores and maximum CT numbers were determined. Agatston scores were compared with the reference at full dose and 70 keV monoE level; deviations (95% confidence interval) < 10% were deemed to be clinically not-relevant.

**Results:**

Averaged over all monoE levels, measured CT numbers deviated from theoretical CT numbers by 6%, 13%, and − 4% for low-, medium-, and high-density CAC, respectively. At 50% reduced dose and 60–75 bpm, Agatston score deviations were non-relevant for 60 to 100 keV and 60 to 120 keV for medium- and high-density CAC, respectively.

**Conclusion:**

MonoE level–specific Agatston score thresholds resulted in similar scores as in standard reconstructions at 70 keV. PCCT allows for a potential dose reduction of 50% for CAC scoring using low monoE reconstructions for medium- and high-density CAC.

**Key Points:**

• *Mono-energy level–specific Agatston thresholds allow for reproducible coronary artery calcium quantification on mono-energetic images.*

• *Increased calcium contrast-to-noise ratio at reduced mono-energy levels allows for coronary artery calcium quantification at 50% reduced radiation dose for medium- and high-density calcifications.*

**Supplementary Information:**

The online version contains supplementary material available at 10.1007/s00330-022-08642-5.

## Introduction

Globally, cardiovascular disease (CVD) is still the number one cause of death [1]. The total amount of calcium in the coronary arteries has been shown to be a superior predictor for a cardiovascular event in the near future, and improves risk prediction when added to conventional risk scores [2, 3].

Traditionally, the amount of coronary artery calcium (CAC) is quantified according to the Agatston methodology, which was developed in the early 1990s on a now obsolete electron beam tomography system [4]. With this method, a CT number specific threshold of 130 Hounsfield units (HU) at a 120-kVp acquisition is used to first discriminate CAC with a minimum density of 100 mg/cm^3^ hydroxyapatite (HA) from surrounding tissue [5]. Next, weighting factors based on the maximum voxel value within CAC lesions are applied, with thresholds of 200, 300, and 400 HU.

Since the introduction of the Agatston scoring methodology, CT scanners have evolved rapidly, with improvements in spatial resolution, temporal resolution, longitudinal coverage, and required radiation dose. Although modern CT scanners have rotation times as fast as 240 ms, the temporal resolution can be further increased by the application of a second X-ray tube and detector in dual-source CT systems [6]. While the temporal resolution in the isocenter of a single-source CT is half the rotation time, the temporal resolution of a dual-source CT is reduced to only a quarter of the rotation time.

Recently, a major new development was introduced in the field of CT: spectral photon-counting CT (PCCT) [7–10]. Whereas conventional CT systems use energy-integrating detectors, PCCT systems use photon-counting detector technology. This enables the detection of photons within certain energy bins, thereby creating an energy-discriminating photon-counting system. Siemens Healthineers has very recently introduced such a system, the NAEOTOM Alpha, which is a dual-source CT scanner with photon-counting detectors. With this system, data can be acquired at a high temporal resolution, while maintaining spatial resolution. Moreover, spectral data is acquired during every scan by both detector arrays, which enables the reconstruction of CT images for virtual mono-energetic (monoE) X-ray sources while maintaining the high temporal resolution of dual-source CT. Thanks to the relatively large increase of X-ray attenuation by calcium when lowering the X-ray energy, calcium contrast is enhanced at reduced monoE levels of 40 to 60 keV compared to the standard CAC protocol with 70 keV reconstructions [11]. We hypothesize that this feature could be used to reconstruct reduced monoE levels from data acquired at a reduced radiation dose by a reduction of the tube current, and with equal CAC contrast-to-noise ratios (CNR) in comparison with the full-dose standard CAC protocol thanks to a balance in the increase in image noise and CAC contrast. However, as CT values are energy dependent, Agatston thresholds adjustments are needed in order to calculate Agatston scores at reduced monoE levels. These thresholds have not been calculated, nor validated, yet.

The primary aim of the current study was, therefore, to determine and validate these adjusted thresholds for monoE images, to yield the same Agatston scores irrespective of the chosen monoE level. The secondary aim was to examine the dose reduction potential of PCCT CAC scans reconstructed at low monoE levels.

## Materials and methods

### MonoE CAC threshold calculation

Theoretical CAC thresholds at different monoE levels were calculated with data from the National Institute of Standards and Technology (NIST) database [12]. For this, the chemical composition of hydroxyapatite (HA) (Ca_10_(PO_4_)_6_(OH)_2_) and its density of 3.15 g/cm^3^ were used [13]. For this compound, the attenuation at 40–190 keV (at 10-keV steps) was calculated with the use of the XCOM NIST database. Furthermore, the attenuation of water and air was calculated at the same monoE levels, to be able to calculate the HU values according to:

$$ \mathrm{CT}\ \mathrm{number}\ \left[\mathrm{HU}\right]=1000\ \frac{\mu_{\mathrm{CAC}}-{\mu}_{\mathrm{water}}}{\mu_{\mathrm{water}}-{\mu}_{\mathrm{air}}} $$

with the attenuation of CAC (*μ*_CAC_) equal to the density-weighted attenuations of water and HA for different mixtures. The CAC threshold of 130 HU at 120 kVp was based on a CAC density of 100 mg/cm^3^ [5]. As the 70-keV reconstruction is the approximation to a standard reconstruction for 120-kVp acquisitions typically used for Agatston scoring, a 130-HU threshold was used for our standard reconstruction. The CAC densities corresponding to a threshold of 200, 300, and 400 HU at 70 keV were determined as well according to the above equation.

### Data acquisition and reconstruction

An anthropomorphic thorax phantom (QRM-thorax, PTW) was scanned on a first-generation dual-source PCCT (NAEOTOM Alpha, Siemens Healthineers, Syngo version VA40A.1.02). A water compartment was placed at the center of the thorax phantom. In this compartment, an artificial coronary artery (diameter 5 mm) was translated by a computer-controlled lever (QRM-Sim2D, PTW) at 0 and 20 mm/s, corresponding to 0 and 60–75 bpm, respectively [14, 15]. The ECG output of the computer-controlled lever was used for ECG triggering to ensure data acquisition during linear motion of the artificial coronary artery [15]. The coronary artery contained three cylindrical calcifications of equal size (1 mm length, 5 mm diameter) but different densities. The HA densities were 196 ± 3, 408 ± 2, and 800 ± 2 mg/cm^3^, i.e., low, medium, and high density, respectively. Phantom dimensions were increased by a fat tissue equivalent extension ring (QRM-extension ring, PTW) to resemble a large patient size [5].

Data was acquired with vendor-recommended sequential CAC protocols at a tube potential of 120 kVp, tube current time product (effective mAs) of 20 mAs (equal to an automatic tube current modulation setting of CARE keV IQ level 16, where CARE keV represents the automatic exposure control of the PCCT system), volumetric CT dose index (CTDI_vol_) of 4.06 mGy, collimation of 144 × 0.4 mm, rotation time of 0.25 s, and a temporal resolution at the isocenter of 66 ms. Incoming X-ray photons were counted within two energy bins, which were predefined by the manufacturer. The data acquisition window was positioned at 60% of the simulated cardiac cycle to ensure acquisition during linear motion of the phantom, without turning points of the robotic arm. To assess the radiation dose reduction potential, additional data was acquired at 50% radiation dose by reducing the tube current time product to 10 mAs. Images were reconstructed at 3 mm slice thickness, 1.5 mm slice increment, Qr36 reconstruction kernel, 220 mm field of view, 512 × 512 matrix elements, filtered back projection, and monoE (Monoenergetic Plus, Siemens Healthineers) levels of 40, 50, …, 190 keV. All acquisitions were performed five times, with manual repositioning of approximately 2 mm translation along the longitudinal axis and 2° rotation around the longitudinal axis of the phantom in between each scan.

### Data analysis

For each CAC density at full dose and 0 bpm, maximum CT numbers from the 5 repetitions were compared to the theoretical CT numbers calculated with data from the NIST database. These maximum CT numbers were, like the Agatston scores, calculated with a previously validated, in-house-developed fully automated quantification method (FQM) written in Python [16]. For this, the theoretical monoE–specific thresholds for both CAC detection and Agatston score weighting factors were added to FQM, where they were used instead of the conventional 130 HU threshold for both CAC detection and quantification. In order to assess the potential for radiation dose reduction with reduced monoE level reconstructions, the CNRs were determined with FQM for all CAC densities, according to:

$$ \mathrm{CNR}=\frac{\left|{\mathrm{Mean}}_{\mathrm{CAC}}-{\mathrm{Mean}}_{\mathrm{Background}}\right|}{{\mathrm{Standard}\ \mathrm{deviation}}_{\mathrm{Background}}} $$

Where all voxels which exceed the CAC threshold were used to calculate the mean CAC CT number, and where the background mean CT number and standard deviation were calculated in a region of interest (50 × 50 mm^2^) in the water compartment.

All Agatston scores were compared with the reference at full dose and a monoE level of 70 keV. Differences (95% confidence interval) in Agatston score with the reference of < 10 were deemed to be clinically not-relevant [17].

## Results

**Fig. 1:**
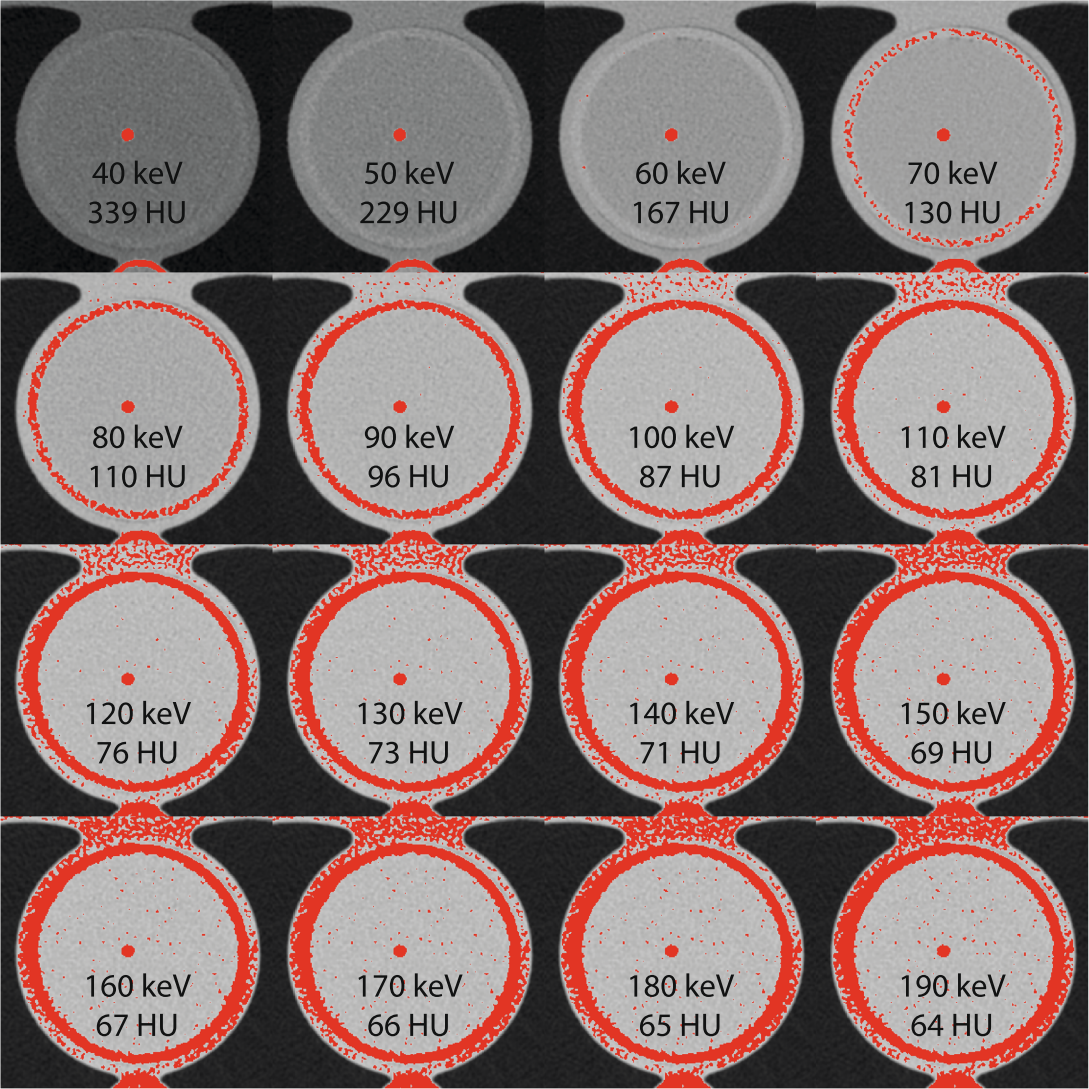
Example images for the medium-density CAC for all monoE levels as indicated in the images. For each monoE level, the theoretical CAC threshold (as shown in the figure) is used to indicate all voxels which exceed this value

**Fig. 2:**
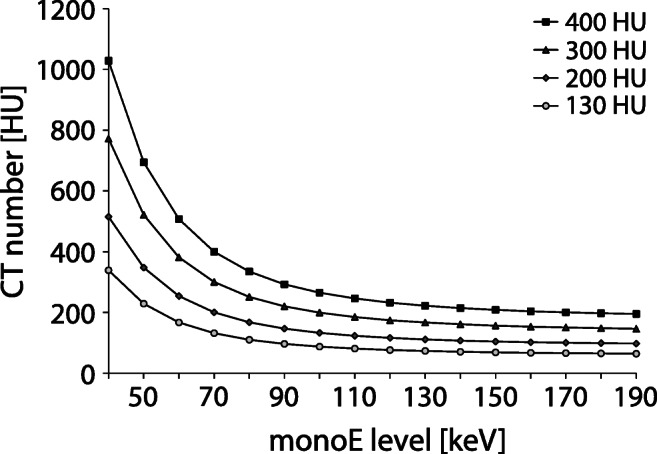
Thresholds for CAC discrimination and Agatston score weighting factors for different monoE levels, with respect to the conventional thresholds of 130, 200, 300, and 400 HU at the reference of 70 keV

Whereas the mass attenuation coefficients of air, water, and pure hydroxyapatite at monoE levels of 130 keV and larger are very similar and in the range of 0.10 to 0.16 cm^2^/g, at lower monoE levels, the mass attenuation of HA strongly diverges from the mass attenuation of water and air (Supplemental Figure 1). Application of the Agatston score threshold on monoE level reconstructions of 40 to 190 keV is shown in Fig. 1. All four Agatston score thresholds for these monoE levels are shown in Fig. 2.

**Fig. 3:**
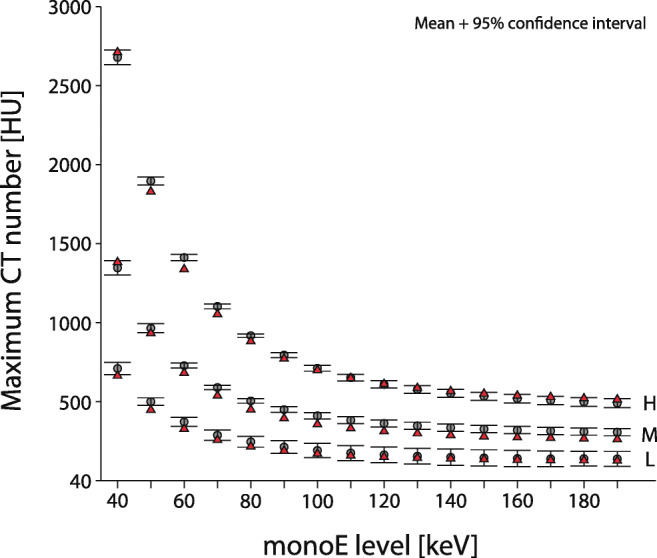
Comparison of the theoretical CT number (red triangles) with the measured CT number (mean and 95% confidence interval plots) for the static low (L)–, medium (M)–, and high (H)–density CAC for all monoE levels

In Fig. 3, a comparison between the theoretical CT numbers for the known CAC densities and the measured maximum CT number per CAC density is shown. Averaged over all monoE levels, measured CT numbers deviated from the theoretical CT numbers by 6%, 13%, and − 4% for the low-, medium-, and high-density CAC, respectively.

**Fig. 4:**
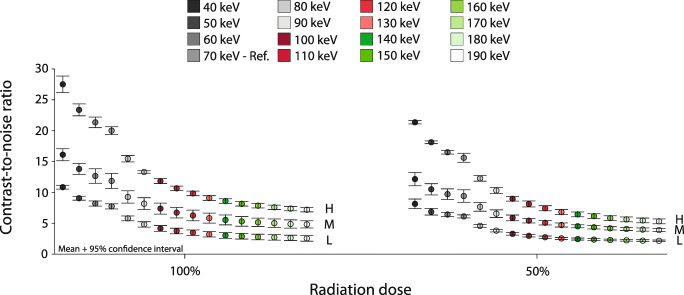
Contrast-to-noise ratio for different monoE levels from 40 to 190 keV (left to right mean and 95% confidence interval plots) for the static low (L), medium (M), and high (H) CAC at 100% (left) and 50% (right) dose

For each CAC density, CNR increased at reduced monoE levels (Fig. 4). The median (total range) CNR at 100% radiation dose and a monoE level of 70 keV was 7.7 (7.4;8.2), 11.4 (10.8;13.2), and 20.0 (19.3;20.7) for the low-, medium-, and high-density CAC, respectively. Virtually the same CNRs at 50% radiation dose were found at monoE levels of 40 and 50 keV for low- and medium-density CAC, while for the high-density CAC, a similar CNR was found at 40 keV only.

**Fig. 5:**
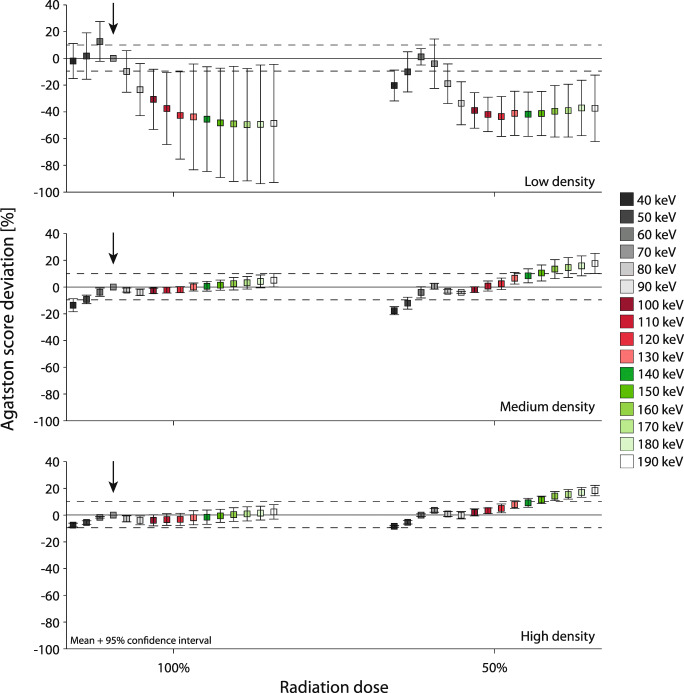
Deviation of the Agatston scores of mono-energetic reconstructions at 40 to 190 keV (left to right mean and 95% confidence interval plots) on PCCT with respect to the reference Agatston scores for static low (top)–, medium (middle)–, and high (bottom)–density CAC, at 100% (left) and 50% (right) dose

For the high- and medium-density CAC, 100% radiation dose static coronary Agatston scores deviated less than 10% from the reference (70 keV, 100% dose) for almost all monoE levels (Fig. 5). Only for the medium-density CAC at 40 and 50 keV, relevant deviations were found. For the low-density CAC, however, relevant deviations were found for all monoE levels. At monoE levels of 40 and 50 keV, mean Agatston score deviations were again non-relevant. However, confidence intervals for these reconstructions overlapped the 10% threshold.

For all calcification densities and monoE levels, 82% and 69% of all combinations led to non-relevant differences in Agatston score for 100% and 50% radiation dose, respectively. Therefore, at 50% reduced radiation dose, an increase in the number of relevant Agatston score deviations was found (Fig. 5). At 50% radiation dose, non-relevant differences in Agatston score deviation with the reference were obtained for 60 keV, 60 to 120 keV, and 40 to 130 keV for low-, medium-, and high-density CAC, respectively.

**Fig. 6:**
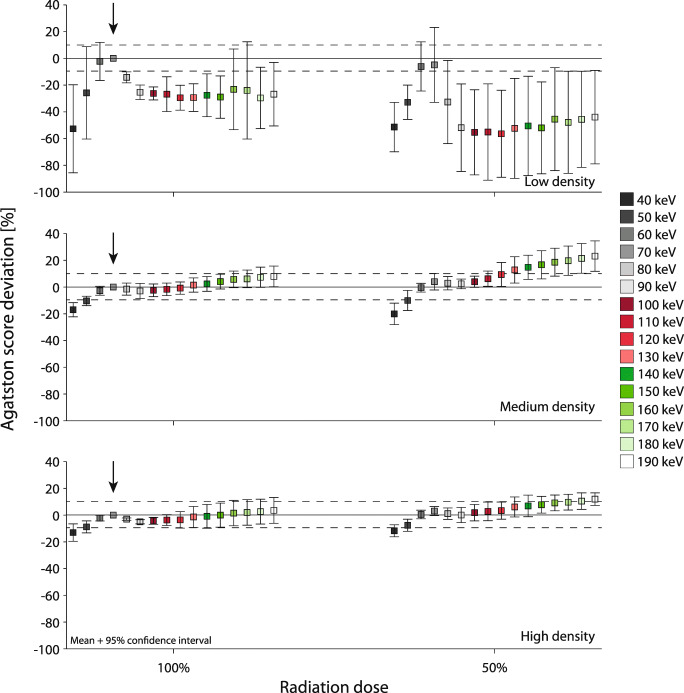
Deviation of the Agatston scores of mono-energetic reconstructions at 40 to 190 keV (left to right mean and 95% confidence interval plots)) on PCCT with respect to the reference Agatston scores for low (top)–, medium (middle)–, and high (bottom)–density CAC at 60–75 bpm, at 100% (left) and 50% (right) dose

The dynamic coronary scan, corresponding to heart rates of 60–75 bpm, showed a similar behavior, although with slightly less non-relevant deviations from the reference Agatston score (Fig. 6). At 50% radiation dose reduction, non-relevant differences in Agatston score with the reference were found for 60 to 100 keV and 60 to 120 keV for medium- and high-density CAC, respectively. For dynamic low-density CAC, non-relevant differences were not shown at 50% radiation dose reduction.

## Discussion

In this study, we determined and applied monoE level–specific Agatston score thresholds for CAC scoring on PCCT. Due to an increased CNR at reduced monoE levels, a potential dose reduction of 50% was found for medium- and high-density CAC with appropriate CAC density–specific monoE levels.

With decreasing CAC density, an increase in the deviations from the reference was found. Especially for the low-density CAC at high monoE levels, large deviations (up to −83%) were shown. These deviations were due to the low CNR levels, in comparison with the reference CNR. Furthermore, while low-density CAC showed a further reduced Agatston score at increased monoE levels, the opposite was found for the medium- and high-density CAC. At increased heart rate, overall Agatston score variability increased, in particular for the low-density CAC. A post hoc power analysis revealed that particularly this low-density category was statistically underpowered (*1 – β*: 0.07) to detect clinically relevant differences. For low-density CAC, the threshold for relevant deviations in Agatston score > 10% may be too strict, especially considering the large number of parameters (including patient size, heart rate, CT system, slice thickness, and CAC quantification parameters) which influence this measurement [15, 16, 18–24].

To the best of our knowledge, this study is the first to calculate Agatston scoring thresholds for monoE images on PCCT. For this, the conventional minimum CAC density of 100 mg cm^−3^ was used [5]. While the current study showed that the increased CNR at reduced monoE levels could be used to reduce the radiation dose, CAC sensitivity with CT could potentially be increased, when the theoretical thresholds are based on a reduced minimum CAC density (< 100 mg cm^−3^). This might especially be of potential interest for the detection of small and/or low-density CAC, given the important role of zero CAC scores for the risk estimation of cardiovascular disease [25]. Moreover, small and/or low-density CAC show reduced CAC scores at increased heart rates [15]. This could result in an erroneous zero Agatston score, when no voxels exceed the conventional primary threshold of 130 HU at a reference tube voltage of 120 kVp.

In the current study, tube current reduction was used to reduce the radiation dose. Many other methods are available to reduce radiation dose for CT CAC assessment, including tube voltage reduction [26]; however, this will also affect the resulting CT numbers. While the current study is the first to assess changes in CT numbers based on monoE reconstruction levels, Nagazato et al previously described a 100-kVp-specific Agatston threshold of 147 HU [27]. Although this increased CAC threshold for reduced energies is in line with our results, a direct comparison is hampered by the fact that the threshold for 100 kVp is based on a polychromatic spectrum, while our theoretical threshold is based on a virtual monochromatic reconstruction.

Our study had some limitations which merit consideration. First, the resulting measured CT numbers showed, on average over all monoE levels, deviations up to 13% from the theoretical values for the used CAC densities. However, many factors apart from image noise influence the measured CT numbers, like the limited spatial resolution and limited accuracy of the reported keV levels. Second, anthropomorphic phantoms were used instead of in vivo measurements for the current study, with artificial CAC–containing coronary arteries and artificial tissue–simulating materials. The densities of the artificial CAC with constant volume were mixtures of HA and so-called solid water. In addition, movement of the coronary artery for the dynamic scans was only in one direction. However, the scan times were relatively short as a result of fast rotation times, whereby the constant linear motion of our phantom was deemed sufficient as a model of the complex in vivo motion of coronary arteries [14]. Also, the mass of the calcifications was in the range which is observed in patients [28]. Finally, only a large patient size resembling phantom was used for the current study. While this phantom study indicates the potential of reduced radiation dose CAC assessment when reduced monoE levels are used, these results should be validated in vivo. For this, CAC assessment can be performed on both standard (70 keV) and reduced monoE level reconstructions of patient scans. For the current study, however, one phantom size was deemed sufficient as Leng et al indicated accurate CT numbers on different monoE level reconstructions for different phantom sizes on a previous prototype PCCT system from the same vendor; therefore, we did not anticipate substantial differences in VMI accuracy across phantom sizes [29]. Third, our CAC contrast calculation was based on all voxels which exceeded the CAC threshold. This approach was chosen because of the small diameter, and therefore a small number of voxels of the calcification. Consequently, the resulting CNR was underestimated compared to what to expect for the known CAC densities. Fourth, our results were validated and applied on a single PCCT system. Although this PCCT system is currently the only clinically available PCCT system which can provide monoE reconstructions at high temporal resolution, additional validation of the proposed Agatston method for monoE reconstruction is needed on other PCCT systems as well.

Overall, virtual monoE images at low energy levels allow for a radiation dose reduction of 50% for medium- and high-density CAC when using energy-specific Agatston score thresholds.

## Supplementary Information

ESM 1 (DOCX 124 kb)

## Abbreviations

CAC: Coronary artery calcium
CNR: Contrast-to-noise ratio
CTDI_vol_: Volumetric CT dose index
CVD: Cardiovascular disease
FQM: Fully automated quantification method
HA: Hydroxyapatite
HU: Hounsfield units
monoE: Virtual mono-energetic
NIST: National Institute of Standards and Technology
PCCT: Photon-counting CT

## Declarations

### Guarantor

The scientific guarantor of this publication is Marcel van Straten.

### Statistics and Biometry

No complex statistical methods were necessary for this paper.

### Methodology

• prospective

• experimental

• performed at one institution
