# Supplementary material for: Coronary calcium scores on dual-source photon-counting computed tomography: an adapted Agatston methodology aimed at radiation dose reduction
Source: Eur Radiol. 2022 Mar 1;32(8):5201–9. doi: 10.1007/s00330-022-08642-5 (PMC9279264; doi:10.1007/s00330-022-08642-5)

Supplemental Figure 1 Mass attenuation coefficients for pure hydroxyapatite, water and air for different monoE levels


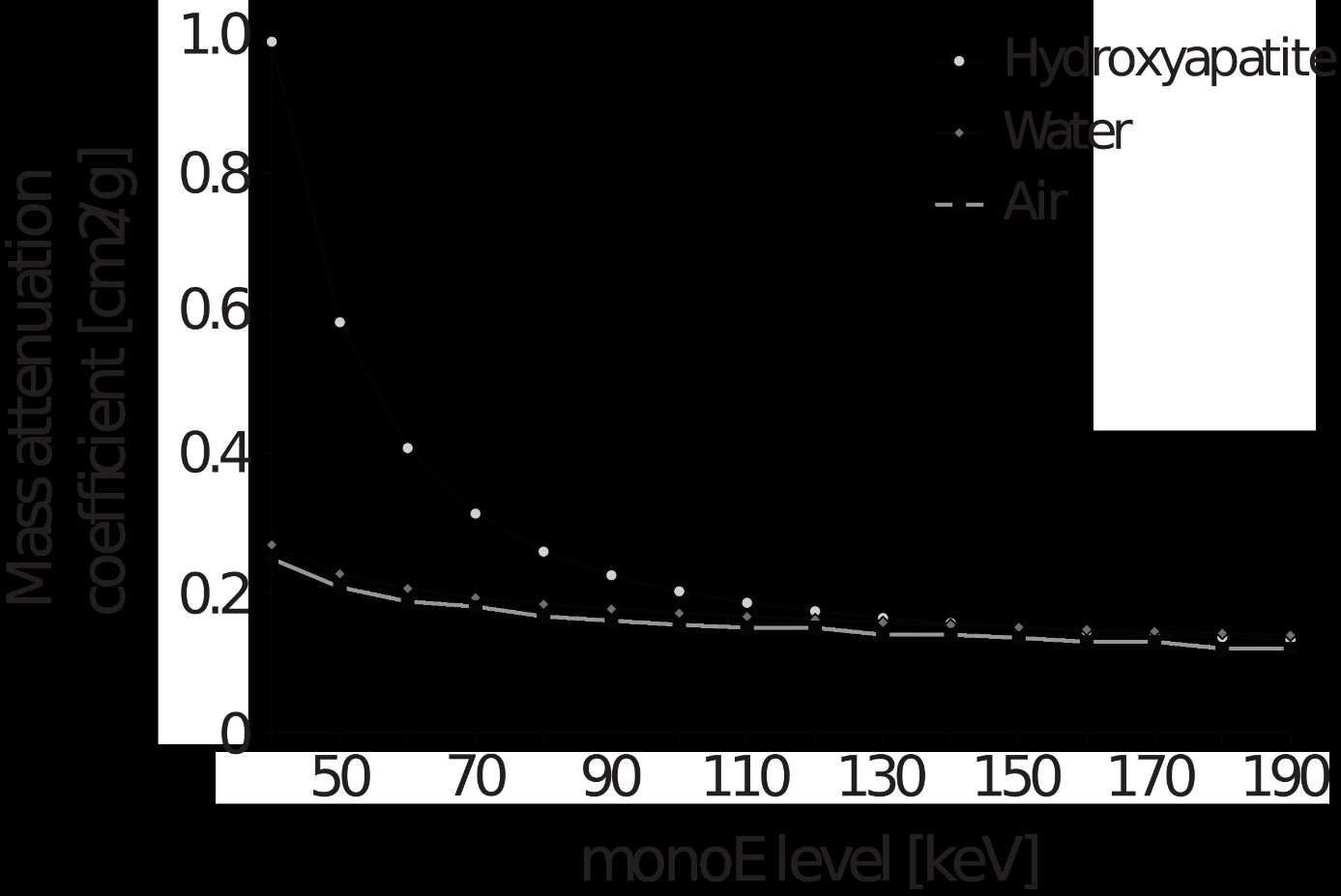

Supplement: Supplementary file 1 — (DOCX 124 kb) [file 330_2022_8642_MOESM1_ESM.docx]
